# Using Failure Mode and Effects Analysis in Improving Nursing Blood Sampling at an International Specialized Cancer Center

**DOI:** 10.31557/APJCP.2021.22.4.1247

**Published:** 2021-04

**Authors:** Anas Haroun, Majeda A AL- Ruzzieh, Najah Hussien, Abdelrahman Masa’ad, Rateb Hassoneh, Ghada Abu Alrub, Omar Ayaad

**Affiliations:** *King Hussein Cancer Center, Al-Jubeiha Amman, Jordan. *

**Keywords:** FMEA, nursing, sampling process, specialized cancer center, King Hussein Cancer Center

## Abstract

**Background:**

The process of blood sampling is considered one of the primary and most common nursing invasive procedures carried out daily. Any failure at any point could have a severe negative impact on patient outcomes.

**Purpose:**

This project aimed to assess and improve the nursing blood sampling process in a specialized cancer center using failure mode and effect analysis (FMEA).

**Methods:**

An observational analytical design of the nursing blood sampling process using FMEA was conducted in King Hussein Cancer Center in Amman, Jordan. Seven steps were conducted, including a review of the blood sampling process, brainstorming potential failures, listing potential effects of each failure mode, assigning a severity rating for each potential effect, assigning a frequency/occurrence rating for each failure mode, assigning a detection rating scale for each failure mode, and calculating the Risk Priority Number (RPN) for each effect.

**Results:**

Eight (out of 28) main critical failure modes with more than 200 RPN were identified in the blood sampling process. Accordingly, five themes were developed to guide the corrective actions. These themes included: process and responsibility modifications, resource and information technology utilization, patients and family engagement, safety culture, and education and training after implementation of the corrective actions. This resulted in a 58 % reduction in the RPN of major failure modes.

**Conclusion:**

Many factors lead to blood sampling errors. A critical focus should be conducted on the preparation phase due to the possible errors that may occur. Proper identification of patients and blood sample tests are the keys to a significant decrease in blood sampling errors.

## Introduction

The process of blood sampling is considered one of the primary and most common nursing invasive procedures carried out daily. Despite the differences between countries, institutions, and individuals, it has multiple phases that include ordering the blood sample, labeling the tubes, preparing equipment, identifying and preparing the patient, selecting the site, taking the sample, filling the tubes, preparing the samples for transportation, and transporting the samples (Jain et al., 2019; Nikolac et al., 2013; World Health Organization, 2010).

Any failure at any point in this process could have a severe negative impact on patient outcomes, from misdiagnosis, potential miss-transfusion, patient discomfort in obtaining a new specimen, delays in treatment, and improper utilization of expensive resources (Bolton-Maggs et al., 2015; Frietsch et al., 2017; Kaufman et al., 2018; World Health Organization, 2010). Frietsch et al., (2017) indicated that around 18% of critical incident reports are blood sampling errors, while Kaufman et al., (2018) showed that the overall adjusted mixed-up error rates were one per 10,110 samples and one per 35,806 samples at sites that used manual patient identification and electronic identification, respectively.

Many factors may lead to blood sampling errors. These factors include incorrect sample or patient identification, wrong sample labeling, inadequate training and education, lack of process standardization, inappropriate equipment and suppliers, lack of proper patient engagement, limited technological solutions, and staff overload and interruptions (De la Salle, 2019; Forest et al., 2017; Frietsch et al., 2017; Kaufman et al., 2018; World Health Organization, 2010).

For these reasons, adopting a proactive approach to risk management is recommended to ensure a high level of quality and patient safety (Joint Commission, 2013). Failure mode and effect analysis (FMEA) is considered as a proactive analysis tool for critical and high-risk processes and focuses on system design. FMEA utilizes unique information to ease identifying the priorities of any improvement actions by taking feedback from the experience and knowledge of frontline clinicians, including clinical nurses, to recognize approaches in processes that fail or potentially fail. It is a functional methodology conducted by a multidisciplinary team and includes many steps (10 steps) starting by reviewing the process and finishing by evaluating the impact of changes (Jain, 2017)

Many studies show the role of FMEA in reducing the risk of nursing care in different areas such as in preoperative (Kim and Lee, 2016), medication administration (Jain, 2017), and blood transfusion processes (Dehnavieh et al., 2015; Najafpour et al., 2017). Kim and Lee (2016) indicated that FMEA prevents negligent accidents in preoperative preparatory procedures such as operation cancellation and preparation omission. Jain (2017) showed its role by a 60% reduction in the Risk Priority Number (RPN) of prioritised failure modes.

Upon reviewing previous studies, no published studies show the role of FMEA in reducing nursing blood sampling, especially in cancer settings, where patient care is complicated. For this reason, this study aimed to assess and improve the nursing blood sampling process in a specialized cancer center using FMEA.

## Materials and Methods

*Setting*

The project was conducted in King Hussein Cancer Center (KHCC) in Amman, Jordan, from September 2019 to March 2020. KHCC is a not-for-profit organization that provides comprehensive cancer care for Jordanian and international patients with a capacity of 350 beds and 1,200 nurses. 

Yearly, KHCC treats more than 7000 new patients. In 2019, KHCC earned a Magnet® program recognition to be the first hospital in Jordan with this recognition. The nursing department’s total number of events related to blood sampling significantly increased from Jan 2018 until Aug 2019. These events were investigated, and direct administrative corrective actions were taken. However, these events recurred, making us think more effectively in applying a comprehensive review for the process and identifying the risky areas.

*Design*

An observational analytical design of the nursing blood sampling process was conducted in all nursing inpatient units. This design is commonly used to assess and evaluate current situations or processes aiming to improve them,

*Data Management*

FMEA was utilized to assess and evaluate the nursing blood sampling process. This methodology was selected because it is considered a valid process to reduce the risk in different nursing processes (Jain, 2017; Kim and Lee, 2016; Najafpour et al., 2017). Accordingly, the 7-steps for conducting FMEA were utilized (Jain, 2017). See Table 1.

*Formulating the FMEA team*

A multidisciplinary team was formulated consisting of 7 expert members from different areas such as a nurse manager, nursing quality, clinical nurses, quality coordinators, and a health informatics supervisor. 

All members were either involved directly in blood sampling, had a good experience in the blood sampling process, or/and had experience in applying quality tools. After assembling the team, educational and training lectures regarding FMEA were provided to increase the team’s knowledge about this project. 

*Reviewing of the blood sampling process*

**Figure 1:**
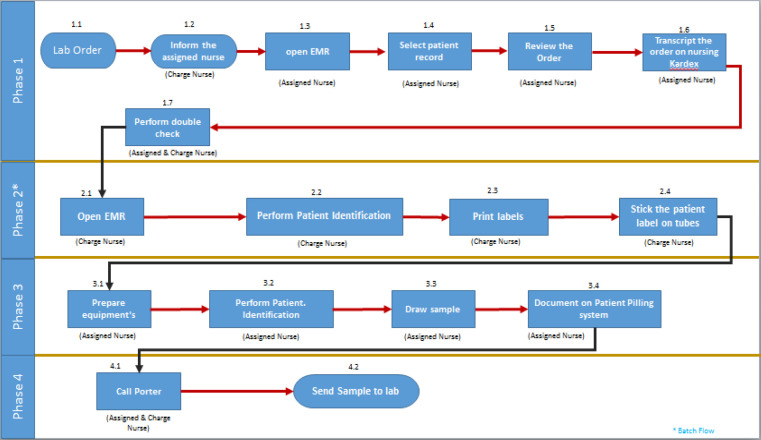
Process Mapping for Nursing Blood Sampling before Interventions

The team developed a process map, which used flowcharts to illustrate the flow of a process, proceeding from the most macro perspective to the level of detail required to identify improvement opportunities. The team collected the required data to develop the process map by arranging regular visits to the relevant nursing unit and reviewing process inputs with their end-users. Then, a business process modeler (Blue Works live^®^) was used to develop the process flow map for the blood sampling process, as illustrated in Figure 1. 

Team members performed an extensive analysis regarding the current flow map. Many concerns and considerations on the current flow map were noticed: the prolonged process that had 17 critical steps for each sampling process; overlapped and repeated process (area covered by multiple people or things, or a situation in which multiple people share responsibility); lack of alignment with relevant policies and procedures; minimal control level at several process steps, so the error was not detected at an early-stage; and fragmented interaction between systems and staff (multi exit-entry on the system). 

*Potential Failures, Causes, Consequences Identification*

At each process step in the process map, brainstorming and focus group were conducted by the team to identify where the errors could have occurred (failure mode) and why the process could fail (failure causes), and what were the consequences of each failure (failure effects). The causes were classified into nine factors (institutional, organization and management, work environment, team, individual staff member, task, and patient factors) based on the approved model developed by the United Kingdom (UK) National Health System (Thomas, 2003). See Table 2

*Severity, Frequency, and Detection Scoring*

The team members scored each failure mode from one to ten according to cause frequency, cause detectability, and consequence severity. “Ten” indicated that the severity and frequency are dangerously high, and its probability of detection was absolutely uncertain. In contrast, “one” indicated the severity and frequency were nonexistent, and its probability of detection was almost certain (Institute for Healthcare Improvement, 2020).

The team’s scoring was individually conducted, followed by a group discussion to determine final scores based on the approved scoring scales that were developed by the Institute of Healthcare Improvement (Institute for Healthcare Improvement, 2020).. 

*Calculating the RPN*

For grading each failure mode, the RPN was calculated for each mode before implementing corrective actions. RPN multiplied the three values (severity x frequency x detection) to prioritize the failure modes (Institute for Healthcare Improvement, 2020). Corrective actions were developed and implemented for each failure mode that scored more than 200 in the initial RPN results. The difference (%) between initial RPNs and RPNs-minus-post-implementation was calculated to measure the improvement. 

*Permission and Ethical Consideration*

Before starting FMEA, the approval for conducting this project was taken from the chief nursing officer. Moreover, the ethical approval was taken from the Institutional review board (IRB) (20 KHCC 130) in KHCC to publish this study.

## Results

*Initial RPNs*

Many failure modes (29 failure modes) with many causes and effects were identified in all steps of the blood sampling process. However, eight main failure modes scored more than 200 in the initial RPN results and were identified as high risk and unacceptable failures, which was around 27.5% of all failure modes. See table 3.

Around 50% of high-risk and unacceptable failure modes were in the ordering phase, including missed test, informing wrong information, wrong patient, and wrong test; 37.5% were in the preparation phase, including the wrong blood in the tube, wrong tube, and wrong test; and 12.5% were in the sampling phase including the wrong patient. The root causes for unacceptable risk failure modes were organization and management factors (50%), work environment (41.6%), individual member factors (37.5%), and team factors (8.3%).

*Intervention*

Accordingly, around five intervention themes were developed to decrease the risk of errors in the blood sampling process. These themes included process and responsibility modifications, resource and information technology utilization, patients and family engagement, safety culture, education, and training; Table 3 summarizes each theme’s interventions, knowing that these interventions were selected based on many previous studies that showed significant reduction in errors and contributing risk factors (Abuseif et al., 2018; Al-Ruzzieh and Ayaad, 2020; Al-Ruzzieh et al., 2020; Ayaad et al., 2019; de Mel et al., 2017; Ning et al., 2016; Rosenbaum and Baron, 2018; Rudrappan, 2019; Sharikh et al., 2020; Stout and Joseph, 2016).

*Process and Responsibility Modifications*

**Figure 2:**
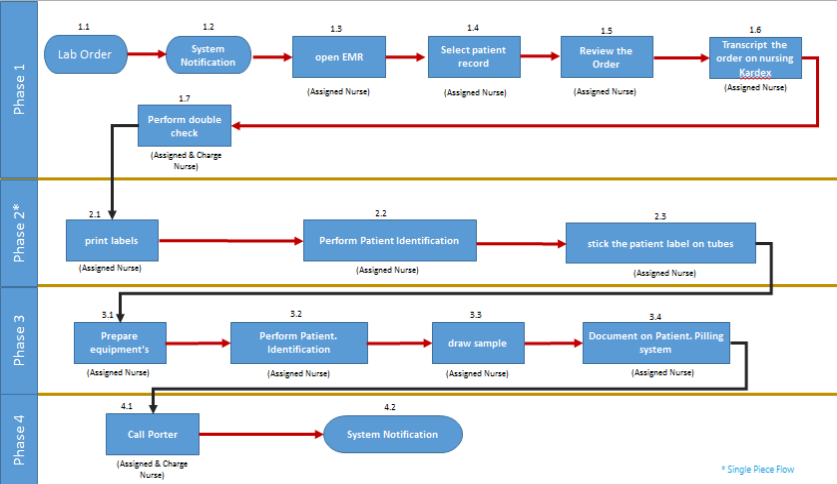
New Process for Blood Sampling after Redesigning

As Figure 2 shows, the blood sampling process was standardized around a new process (‘’single piece flow’’) in which only one patient with one set of patient labels were handled at a time, and a second patient was not phlebotomized until the first patient’s blood samples were prepared. This process aimed to decrease the possibility of sampling errors (wrong patients), especially when a nurse performed the sampling process for several patients at a time. This intervention targeted the missed test, and wrong patient and test failure modes

Moreover, the process was modified and redesigned, aiming to decrease the risk of errors during the sampling process and workload. The assigned nurses became responsible for following and preparing blood sample orders in the morning cycle and printing the labels to decrease charge nurses’ workload. However, assigned nurses would verify the process with the charge nurses. This intervention targeted the missed test and informing wrong information failure modes.

*Resource and Information Technology Utilization*

To ensure the clarity of the stickers’ information, only the required information for the proper patient and test identification was determined. Moreover, a new report was designed in the electronic medical records in order to ease the nursing follow up and review of pending blood sample orders. This report could be generated at any time and for any period of time by the assigned nurses and charge nurses. This intervention targeted the missed test, and wrong patient and test failure modes.

Moreover, the assigned nurses were instructed to review the need for blood samples daily to decrease the possibility of missing blood samplings, especially for those ordered for several days. One printer in each nursing station was assigned for label printing. This process decreased the risk of choosing the wrong printer and eliminated the possibility of having labels printed by other staff in a different section. For this reason, this intervention targeted the wrong patient and test failure modes.

*Patients and Families Engagement*

Patients and their families were engaged during the sampling process by educating them about the sampling process and the importance of their engagement in the process. The nurses were instructed to ask the patients’ preferences before sampling, such as the preferred site for sampling. This intervention targeted the wrong patient mode.

*Safety Culture*

To ensure safety culture in the blood sampling process, the nurses were instructed and encouraged to initiate the event report and near-miss error if any errors related to blood samplings occur or were likely to occur and reflect on their experience. Moreover, new indicators, including “Blood Sampling Mixed Up” and “Nursing Blood Sampling Errors,” were utilized to track sampling errors, which are reported every quarter using external benchmarks.

Good catch award for blood sampling errors was activated. Regular quality rounds were conducted to ensure proper implementation of best practices, including proper patient identification. These interventions targeted all failure modes.

*Education and Training*

The nurse educators trained the nurses on how they can utilize the new process in their practice for new and senior nurses. A simulation lab was utilized for training the nurses and updating their competencies with the blood sampling process. Finally, a Nursing Guideline in Phlebotomy /Reanimation was developed to instruct all nurses in the hospital about the best practices in blood sampling. These interventions targeted all failure modes.

*RPNs- Post-implementation*

After implementing the corrective actions, an average of 58% reduction in the RPN of major failure modes was seen. Moreover, the incidence of blood sample mix-up was decreased by 70% after three months of implementation. Please see table 3.

**Table 1:** General Steps for Conducting Failure Mode and Effect Analysis

Step	Description
1	Reviewing of blood sampling process
2	Brainstorming potential failures
3	Listing potential effects of each failure mode
4	Assigning severity rating for each potential effect
5	Assigning frequency/occurrence rating for each failure mode
6	Assigning detection rating scale for each failure mode
7	Calculating Risk Priority Number (RPN) for each effect

Source, Jain (2017)

**Table 2:** Classification of Root Causes Analysis Results

Cause	Description
Institutional	Regulatory context, Medicolegal environment
Organization and management	Financial resources and constraints, Policy standards and goals, Safety culture and priorities
Work environment	Staffing levels and mix of skills, Patterns in workload and shift Design, availability, and maintenance of equipment, Administrative and managerial support
Team	Verbal communication, Written communication, Supervision and willingness to seek help, Team leadership
Individual staff member	Knowledge and skills, Motivation and attitude, Physical and mental health
Task	Availability and use of protocols, Availability and accuracy of test results
Patient	Complexity and seriousness of condition Language and, communication Personality and social factors

Source, Thomas (2003)

**Table 3:** Summary of Failure Mode and Effect Analysis- Critical Results (RPN ≥200)

Process	Failure Modes	Causes	Effects	RPN1	RPN2	%
Ordering Phase	Missed test	Lack of adequate nursing follow up (organization and management, and Work environment factor)	Delay in treatment plan	200	90	55%
		Phyiscans or nurse incharge did not inform the assigned nurse (Team factor)		240	90	63%
		No systematic process to follow-up the pending lab test (organization and management factor)		245	110	55%
		Incharge- high workload (Work environment and Individual staff member factors)		350	210	60%
	Informing wrong inforamtion	Phyiscans or nurse incharge informs the assigned nurse wrong information (Team factor)	Wrong lab results lead to wrong treatment	420	80	81%
		Incharge- high workload (Work environment factor)		350	210	60%
	Wrong patient	Improper patient identification (Work environment and Individual staff member factor)		288	90	69%
		High workload (Work environment factor)		350	210	60%
	Wrong test	Improper patient identification (Work environment and individual factor)		200	90	55%
		High workload (Work environment and Individual staff member factor)		350	210	60%
Prepartion phase	Wrong patient in tube	Improper patient identification (organization and management factor)	Wrong lab results lead to wrong treatment	240	110	54%
		Printing labels for all patients once, without cutting labels for each patient (organization and management factor)		441	130	71%
		Wrong or extra label printing (organization and management factor)		288	90	69%
		Improper chart review and verification (Work environment and Individual staff member factors)		225	110	51%
		Unclear information on the stickers due to small size of written information (organization and management factor)		225	90	60%
		A lot of information in the stickers (organization and management factor)		245	80	67%
		Unnecessary motion/rework and increase the risk for interruptions during blood sampling process (organization and management factor)		245	120	51%
		There is no visualized material to instruct the nurses about preparing blood samples ( organization and management, and Individual staff member factors)		225	90	60%
	Wrong tube	Putting lable in wrong tube (Individual staff member factors)	Wrong lab results lead to wrong treatment	441	170	61%
		Unnecessary motion/rework and increase the risk for interruptions during blood sampling process (organization and management factor)		245	120	51%
	Wrong test	Selecting wrong test in the system (Work environment and individual staff member factors)	Wrong lab results lead to wrong treatment	225	150	33%
		Unnecessary motion/rework and increase the risk for interruptions during blood sampling process (organization and management factor)		225	120	47%
Sampling Phase	Wrong patient	Improper patient identification before sampling (Work environment and individual staff member factors)	Wrong lab results lead to wrong treatment	220	110	50%
		Starting sampling process for many patient at one time (organization and management factor)		260	90	65%

**Table 4:** Interventions to Improve the Process of Nursing Blood Sampling Process

Theme	Intervention	Targeted Failure Modes
Process and Responsibility Modifications	The assigned nurse becomes responsible for many steps in the preparation phase (2^nd^ phase)	Missed test and Informing wrong information
	Single Piece Flow was utilized instead of batch Flow.	Missed test, and Wrong patient and test
Resource and Information Technology Utilization	A new report was designed in the electronic medical records to determine pending blood sampling orders	Missed test, and Wrong patient and test
	Using one printers in each nursing station for printing the labels	Wrong patient and test
Patients and Families Engagement	Patient and family education about sampling process and time and important of their engagement in the process to prevent errors and ensure providing care according their preferences	Wrong patient
Safety Culture	Reporting the event report and near miss error related to blood sampling	All failure modes
	Good catch award	
	Tracking the errors through adoption of new indicators named “ Blood sampling mixed up” and “ Nursing Blood sampling Errors”.	
	Regular nursing quality rounds	
Education and Training	Providing nursing education about the new process for new and old nurses.	All failure modes
	Training nursing using simulation lab approach.	
	Nursing Guideline in Phlebotomy /Reanimation was developed	

## Discussion

The project was conducted as a proactive risk assessment in a specialized cancer center in Amman, Jordan, using the FMEA methodology to assess and improve the nursing blood sampling process based on many interventions mentioned in previous studies (Abuseif et al., 2018; Al-Ruzzieh and Ayaad, 2020; Al-Ruzzieh et al., 2020; Ayaad et al., 2018; Ning et al., 2016; Rudrappan, 2019; Sharikh et al., 2020) .

Most blood sampling projects have been performed with a reactive approach, especially after a catastrophic event. In this project, a proactive approach was used using FMEA to identify the blood sampling process’s potential failures and their causes and effects and suggest improvement actions. A proactive approach, including the FMEA approach, could avoid the main adverse outcome in health settings ( Jain, 2017; Kim and Lee, 2016; Najafpour et al., 2017).

Around 27.5% of identified failure modes were considered unacceptable modes without any appropriate controlling system. This rate is considered high compared to similar projects (Dehnavieh et al., 2015; Jain, 2017; Kim and Lee, 2016; Najafpour et al., 2017). Around 50% of high-risk and unacceptable failure modes were in the ordering phase; 37.5% were in the preparation phase, and 12.5% were in the sampling phase, including the wrong patient. The ordering phase is the first and most crucial step since every other step depends on this process’s results. 

The root causes for unacceptable risk failure modes were organization and management factors (50%), work environment (41.6%), individual member factors (37.5%), and team factors (8.3%). Organization and management factors were related to a lack of awareness of safety issues and policies, leading to inadequate staffing levels, and increased staff workload. Work environment factors were related to fatigue due to heavy workloads, inadequate administrative support, and limited access to essential equipment which led to reduced time with patients. Individual member factors were related to long-term stress and fatigue and lack of knowledge or experience. These factors significantly impacted the failure to monitor and observe patients, loss of patient information, incorrect assessment, delay in diagnosis and treatment, deviation from or use of the incorrect protocol, and wrong treatment given (Thomas, 2003).

Based on the initial RPN results, many interventions were conducted. Previously conducted studies approved these interventions in enhancing the nursing work environment, quality, and safety of nursing care by using adequate technology such as well-designed electronic medical records, computer-assisted barcode system, automated sample labeling, delta checks, and electronic identification system (Al-Ruzzieh et al., 2020; Ning et al., 2016; Rosenbaum and Baron, 2018; Sharikh et al., 2020); redesigning the process; structured quality and administrative rounds (Ayaad, et al., 2019); proper utilizing of resources; ensuring high nursing autonomy (Abuseif et al., 2018; Al-Ruzzieh and Ayaad, 2020); adequate staff engagement, education and training; and patient engagement (Ayaad et al., 2019; de Mel et al., 2017; Rudrappan, 2019; Stout and Joseph, 2016). 

There was a significant (58%) reduction in the RPN-post intervention results and incidence of blood sampling errors were reduced by 70% after the implementation of corrective actions.

*Limitations*

FMEA is a time-consuming process and requires a specialized team with good experience to analyze and be very familiar with the blood sampling process. It decreases the risk of errors, but it does not eliminate them. For these reasons, a proper action plan should be developed and implemented. Accordingly, proper engagement with frontline staff should be conducted in performing this analysis and developing and implementing the required action plan.

In conclusion, many factors lead to blood sampling errors. A critical focus should be conducted on the preparation phase due to the many possible errors that may occur at this step. Proper identification of patients and blood sample tests are key to a significant decrease in blood sampling errors. A clear guideline should be established to standardize the process. However, proper compliance is required. Policies and procedures are in place; adherence to them is required. FMEA is a continuous and multiphase proactive risk assessment tool. The suggested actions should be utilized with a clear definition of responsibilities. This analysis reduces errors and builds a team to perform the required improvements from a different perspective without adding any additional financial burden on hospitals.

Finally, the results support the facts indicating that the prevention of errors is a cost-effective strategy compared to handling the errors. Moreover, the results confirm FEMA’s applicability in reducing errors in a healthcare setting.

## Author Contribution Statement

The authors confirm contribution to the paper as follows: study conception and design: Anas Haroun, Majeda A AL- Ruzzieh, Rateb Hassoneh, Ghada Abu Alrub; data collection: Najah Hussien, Abdelrahman Masa’ad, Rateb Hassoneh, Ghada Abu Alrub; analysis and interpretation of results: Anas Haroun, Majeda A AL- Ruzzieh, Najah Hussien, Abdelrahman Masa’ad, Omar Ayaad, Rateb Hassoneh; draft manuscript preparation: Omar Ayaad. All authors reviewed the results and approved the final version of the manuscript. 
